# Phytochemical Characterization and Toxicity Activity of *Tradescantia pendula* Extracts

**DOI:** 10.3390/molecules31173000

**Published:** 2026-08-27

**Authors:** Rubi Esmeralda González Campos, Elizabeth Bautista Rodríguez, César Emmanuel Ale, Solón Javier Garcés Eisele, Luis Ricardo Hernández, Zaida Nelly Juárez

**Affiliations:** 1 Faculty of Molecular Engineering and Sustainable Technologies, Deanship Engineering, Universidad Popular Autónoma del Estado de Puebla (UPAEP), Sur 21, 1103 Barrio de Santiago, Puebla 72410, Mexico; rubiesmeralda.gonzalez@upaep.edu.mx (R.E.G.C.); solonjavier.garces@upaep.mx (S.J.G.E.); 2 Puebla State Council for Science and Technology (CONCyTEP), Boulevard Atlixcáyotl 1101, Reserva Territorial Atlixcáyotl, Concepción las Lajas, Centro Integral de Servicios (CIS), Puebla 72190, Mexico; 3 Area of Chemistry, Department of Sciences, Universidad Popular Autónoma del Estado de Puebla (UPAEP), Sur 21, 1103 Barrio de Santiago, Puebla 72410, Mexico; 4 Multidisciplinary Laboratory in Biomedicine, Biotechnology and Integrative Bioinformatics Applied to Health (LAMB3IS), Faculty of Health Sciences, Autonomous University of Tlaxcala (UATx), Zacatelco 90750, Mexico; ebautistar@uatx.mx; 5 Institut de Pharmacologie et de Biologie Structurale (IPBS-CNRS), 31077 Toulouse, France; 6 Faculty of Agronomy, Zootechnics and Veterinary Medicine, National University of Tucuman, Avenida Kirchner 1900, San Miguel de Tucumán 4000, Tucumán, Argentina; cesar.ale@faz.unt.edu.ar; 7 Department of Chemical Biological Sciences, Universidad de las Américas Puebla, Ex Hacienda Santa Catarina Mártir S/N, San Andrés Cholula, Puebla 72810, Mexico

**Keywords:** *Tradescantia pendula*, antimicrobial activity, GC-MS, phytochemical screening, *Artemia* model, cytotoxicity, medicinal plants

## Abstract

*Tradescantia pendula* is a medicinal plant traditionally used in Mexico to treat inflammatory and infectious conditions. This study evaluated the phytochemical composition, antimicrobial activity, toxicity, and cytotoxicity of *T. pendula* extracts. Plant material was sequentially extracted using hexane, chloroform, and methanol, followed by phytochemical screening, antimicrobial evaluation against clinically relevant bacterial strains, toxicity assessment using the *Artemia salina* model, cytotoxicity analysis in HaCaT and U937 cell lines, and GC–MS characterization. Phytochemical screening of the chloroform extract revealed the presence of flavonoids, tannins, triterpenes, and glycosides. Antimicrobial activity was observed mainly against *Escherichia coli* and *Pseudomonas aeruginosa*, with bacterial viability reduced by up to 95.2%. Toxicity assays in *Artemia salina* demonstrated marked biological activity, with LC50 values of 63.54, 23.12, and 23.05 μg/mL for the hexane, chloroform, and methanol extracts, respectively. Cytotoxicity assays revealed cell type-dependent responses, with U937 monocytic cells exhibiting greater sensitivity than HaCaT keratinocytes. All three extracts produced non-monotonic concentration–response profiles, with enhanced metabolic activity at intermediate concentrations and substantial decreases in cell viability at lower concentrations. GC–MS analysis revealed lipophilic metabolites, including phytol-related compounds and fatty acid esters. These findings suggest that *T. pendula* is a promising source of bioactive metabolites with antimicrobial and cytotoxic properties, supporting its ethnopharmacological relevance and potential pharmacological applications.

## 1. Introduction

Traditional medicine in Mexico is a form of healthcare deeply rooted in the country’s indigenous and rural cultures, integrating botanical knowledge, rituals, and empirical diagnoses. This type of medicine, recognized by the World Health Organization (WHO), remains fundamental in communities where access to conventional healthcare services is limited or non-existent [1]. In Mexico, an estimated 60% of the population has used herbal remedies at some point, reflecting their cultural and therapeutic importance [2]. The country boasts a wealth of biological diversity, resulting in a vast inventory of plant species with medicinal uses. One of these is *Tradescantia pendula* (synonym: *Tradescantia zebrina*), a plant in the family Commelinaceae, commonly known as ‘amor de hombre’ (man’s love) or ‘cucaracha morada’ (purple cockroach).

*Tradescantia pendula* (Commelinaceae) is a perennial creeping herb characterized by succulent, trailing stems that readily root at the nodes. It has simple, alternate leaves with sheathing bases and produces pink to purple flowers arranged in small terminal inflorescences. The species is widely distributed throughout tropical and subtropical regions and is commonly cultivated as an ornamental plant owing to its attractive foliage [3]. In addition to its ornamental value, this species is widely used in traditional medicine to treat ailments such as inflammation, coughs, respiratory infections, gastrointestinal conditions, and superficial wounds [2,4].

Several species of the genus *Tradescantia* have been used in traditional medicine for centuries in different regions of the world. Depending on the species, various herbal preparations, including aqueous extracts, infusions, and topical applications, have traditionally been used to manage inflammatory disorders, bacterial infections, digestive ailments, kidney diseases, and insect stings. These ethnomedicinal uses have stimulated scientific interest in investigating the phytochemical composition and biological activities of *Tradescantia* species. However, the pharmacological properties and safety profile of *Tradescantia pendula* remain comparatively underexplored, particularly regarding its antimicrobial activity, toxicity profile, and cytotoxic potential [4,5]. In southeastern Mexico, particularly in the state of Tabasco, the plant locally known as “matalí” has traditionally been consumed as a refreshing herbal infusion and used in folk medicine to treat dysentery, inflammatory conditions, infected wounds, and hemorrhages. In addition to their medicinal applications, *Tradescantia* species were widely cultivated as ornamental plants for their attractive foliage [6].

Recent studies have documented that *T. pendula* contains various phenolic compounds and flavonoids, which confer antioxidant, anti-inflammatory, and antimicrobial properties. These findings have sparked scientific interest in validating its traditional uses through phytochemical, pharmacological, and toxicological studies [7]. Despite its widespread use in traditional medicine, the phytochemical composition and biological activities of *T. pendula* remain insufficiently characterized. Most available studies have focused on identifying phenolic compounds and assessing antioxidant properties, whereas information on its antimicrobial potential, toxicity profile, and effects on mammalian cells remains limited. Furthermore, the contribution of lipophilic metabolites to this species’ biological activity has received comparatively little attention.

Evaluating medicinal plants requires not only characterizing their beneficial biological activities but also assessing their potential toxicological effects. Toxicity and cytotoxicity studies provide essential information on the safety and biological selectivity of plant-derived extracts and help identify promising sources of bioactive compounds. In addition, advances in chromatographic and spectrometric techniques, such as gas chromatography–mass spectrometry (GC–MS), allow the identification of metabolites potentially associated with the biological activities observed in complex plant extracts.

In this context, the present study aimed to evaluate the phytochemical composition and biological activity of hexane, chloroform, and methanol extracts obtained from the stems and leaves of *Tradescantia pendula*. Specifically, we investigated antimicrobial activity against clinically relevant bacterial strains, toxicity in the *Artemia salina* model, and cytotoxicity in HaCaT and U937 cell lines. Additionally, we performed GC–MS analysis to identify metabolites potentially associated with the observed biological activities. This approach supports the scientific validation of the traditional medicinal use of *T. pendula* and provides new information on its pharmacological potential.

## 2. Results

The plant material was cut and dried, yielding 562.45 g (8.44% yield). Table 1 shows the extract yields relative to dry plant material.

In the absence of published data for *Tradescantia pendula*, residual moisture was compared with values reported for *Rhoe discolor*, another member of the Commelinaceae family evaluated under comparable drying conditions. The literature reports that this species can have a moisture content of 50%. García-Varela [8] also reported that *Rhoe discolor* obtained from an aqueous extract had a moisture content of 93.53% of its total fresh mass.

### 2.1. Phytochemical Screening

Table 2 summarizes the results of the preliminary phytochemical screening of the chloroform extract of *Tradescantia pendula*. Alkaloids were not detected, as no precipitate was observed in the corresponding qualitative test, while saponins were also absent because neither stable foam formation nor a violet color change occurred. In contrast, the triterpene assay produced a characteristic red ring, suggesting the presence of triterpenes in the extract. These widely distributed secondary metabolites play essential roles in plant defense, growth, and ecological interactions; however, information regarding their occurrence in members of the Commelinaceae family remains limited, highlighting the need for further phytochemical investigations of this taxon.

The extract formed a precipitate with the gelatine reagent. It can be concluded that, in this species, there is a concentration of tannins produced by the plant, and, given that the precipitate obtained turns green upon addition of ferric chloride, it is inferred that these tannins are of the catechin or condensed type. However, the small amount of precipitate obtained indicates a low tannin concentration in the sample. The qualitative phytochemical analysis of *T. pendula* also confirmed the presence of flavonoids, as positive results were obtained with the Shinoda, Pew, and sodium hydroxide tests. Finally, the Baljet test with picric acid in alkaline solution confirmed the presence of cardiotonic glycosides, as evidenced by the development of an orange coloration (Table 2).

### 2.2. Evaluation of the Antimicrobial Activity of Extracts

The antimicrobial activity of matalí extracts was assessed by testing the chloroform and hexane fractions against various bacterial strains. The chloroform extract exhibited minimum inhibitory concentrations (MICs) of 25,800 μg/mL against *Escherichia coli* 2537 and 404 μg/mL against both *E. coli* 3002 and *Pseudomonas aeruginosa* 3134. In contrast, the hexane extract did not inhibit *E. coli* 2537 or *E. coli* 3002 at the highest concentration tested (103,400 μg/mL), but it showed an MIC of 51,700 μg/mL against *P. aeruginosa* 3134. No antimicrobial activity was detected against *Staphylococcus aureus* QO1 or *S. epidermidis* 13538 under the experimental conditions. Concentrations above these values exceeded the solubility limits of DMSO (Table 3).

### 2.3. Effect on Viability

Results from applying extracts at sublethal concentrations show reduced bacterial viability across different concentrations and extraction solvents. For strain *E. coli* 2537, the control showed a viability of 2.5 × 10^7^ CFU/mL, while treatment with the chloroform extract at 25,800 μg/mL reduced the viable population to 1.2 × 10^6^ CFU/mL, a 95.2% reduction compared to the control. Similarly, for *E. coli* 3002, the control showed a viability of 3.1 × 10^7^ CFU/mL. In contrast, exposure to the chloroform extract at 404 μg/mL reduced viability to 5.3 × 10^6^ CFU/mL, resulting in an 82.9% reduction.

For *Pseudomonas aeruginosa* 3134, the control reached 9.5 × 10^6^ CFU/mL. When exposed to chloroform extract at 404 μg/mL, viability decreased to 3.4 × 10^6^ CFU/mL, representing a 64.2% reduction. Meanwhile, the hexane extract at 51,700 μg/mL decreased to 4.5 × 10^6^ CFU/mL, a 52.6% reduction compared to the control (Table 4).

**Figure 1:**
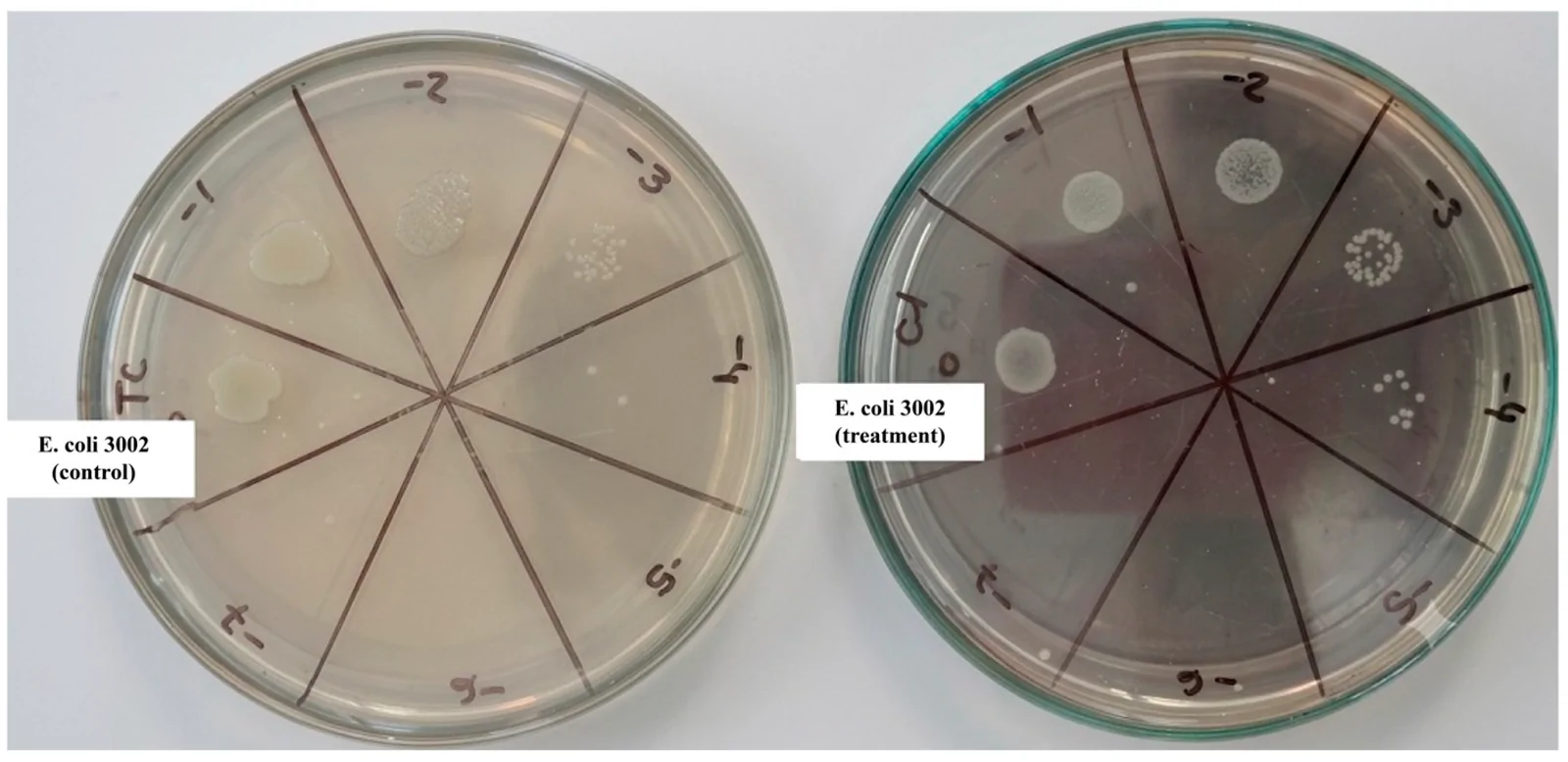
Representative image of the final viability after 24 h of incubation of the microorganisms under study (*E. coli* 3002 in this example) without (control) and with chloroform extract (treatment).

Figure 1 shows a representative image of the test performed.

### 2.4. The LC_50_ Values for Tradescantia pendula Extracts Against Artemia After 24 h of Exposure Are Shown in Table 5

The hexane, chloroform, and methanol extracts exhibited similar toxicity, with the methanol extract showing the lowest toxicity, as indicated by a relatively high LC_50_ value.

The 50% lethality (LC_50_) assay showed significant differences in the acute toxicity of *Tradescantia pendula* extracts toward *Artemia* sp. compared with the control. Although all extracts exhibited toxic activity, the chloroformic and methanolic extracts were the most toxic.

**Table 5:** Toxicity of *Tradescantia pendula* extracts in brine shrimp test.

Extract	LC_50_ (μg/mL)	Confidence Interval (95%)
Hexane	63.54	0–105.1
Chloroform	23.12	0–48.18
Methanol	23.05	0–78.62

### 2.5. Effects of Tradescantia pendula Extracts on HaCaT Cell Viability

**Figure 2:**
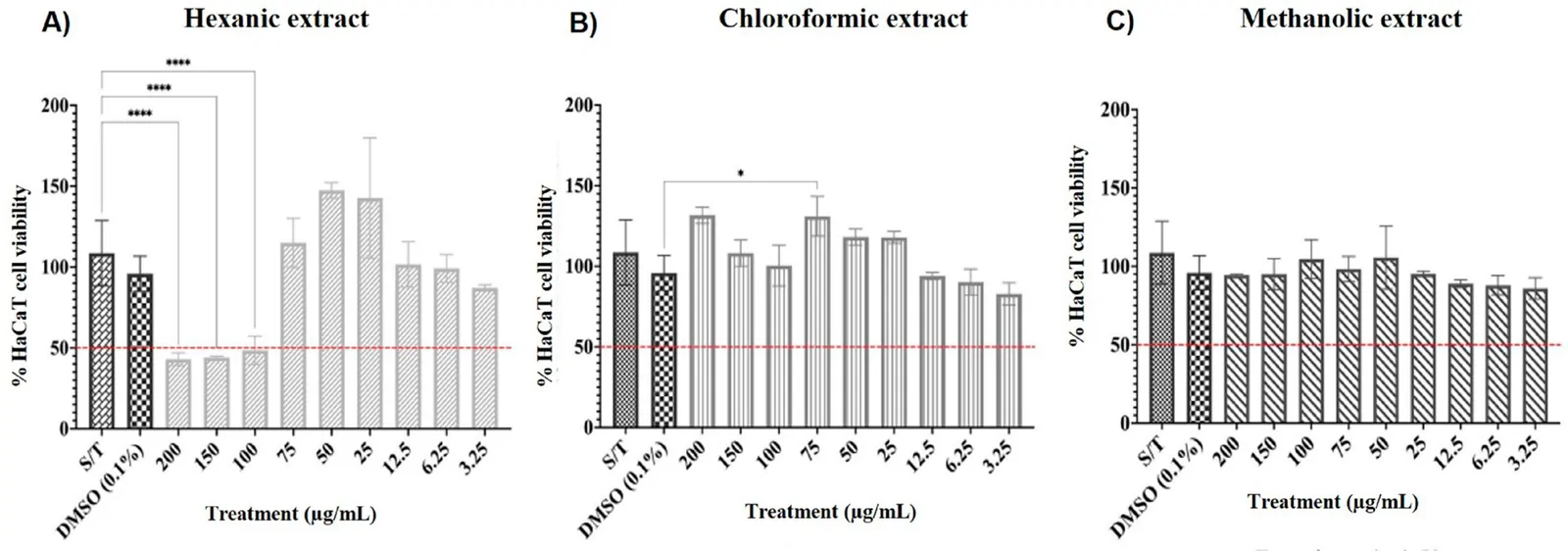
Effect of the hexanic, chloroformic, and methanolic extracts of *Tradescantia pendula* on HaCaT cell viability after 24 h of exposure. Cells were treated with increasing concentrations (3.25–200 μg/mL) of the (**A**) hexanic extract, (**B**) chloroformic extract, and (**C**) methanolic extract. Cell viability was determined by the MTT assay and expressed as a percentage relative to untreated control cells. Data are presented as mean ± SD of three independent experiments. Statistical analysis was performed using one-way ANOVA followed by Tukey’s multiple comparison test. Asterisks indicate significant differences compared with untreated control and vehicle control (0.1% DMSO): *p* < 0.05 (*) and *p* < 0.0001 (****).

The effects of the hexanic, chloroformic, and methanolic extracts of *Tradescantia pendula* on HaCaT cell viability were evaluated after 24 h of exposure using the MTT assay (Figure 2).

The hexanic extract produced the most pronounced effect on HaCaT cells. Metabolic activity decreased significantly at 200, 150, and 100 μg/mL, with cell viability reduced to approximately 40–50% relative to untreated controls (*p* < 0.0001). At intermediate concentrations (75–25 μg/mL), cell viability increased substantially, exceeding 140%, indicating a non-monotonic concentration-response pattern. At lower concentrations (12.5–3.25 μg/mL), viability remained above the cytotoxicity threshold and gradually approached control levels (Figure 2A). In contrast, the chloroformic extract exerted limited effects on HaCaT cell viability.

Cell viability remained above 80% at all concentrations tested, with a modest but statistically significant increase at 75 μg/mL compared with other treatment groups (*p* < 0.05). No concentration reduced viability below 50%, indicating low cytotoxicity under the experimental conditions (Figure 2B). Similarly, the methanolic extract caused minimal changes in cellular metabolic activity, with viability values remaining close to those of untreated controls across the tested concentration range and no statistically significant differences detected among treatment groups (Figure 2C). These results show that HaCaT cell responses depended on the extraction solvent. The hexanic extract had the most pronounced effect on cellular metabolic activity and was the only extract to reduce viability below 50% at higher concentrations, whereas the chloroformic and methanolic extracts exhibited limited cytotoxicity toward keratinocytes.

### 2.6. Effects of Tradescantia pendula Extracts on U937 Cell Viability

**Figure 3:**
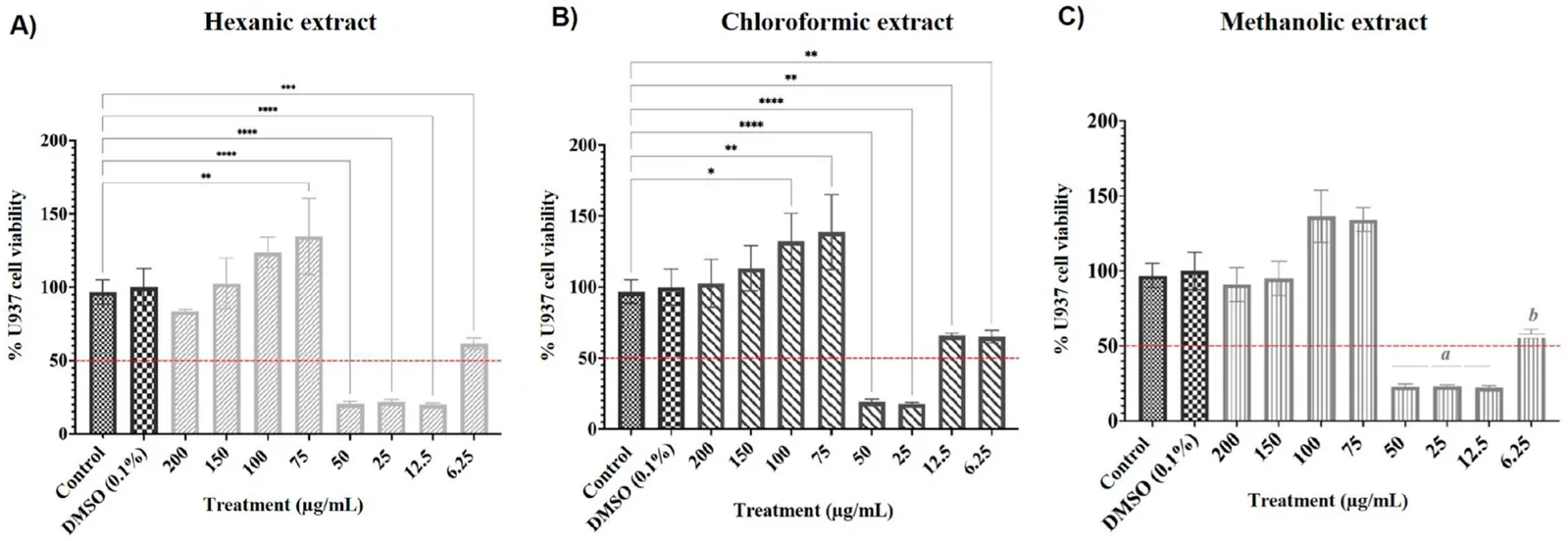
The effect of the hexanic, chloroformic, and methanolic extracts of *Tradescantia pendula* on the viability of U937 cells after 24 h of exposure. The cells were exposed to increasing concentrations (6.25–200 μg/mL) of the (**A**) hexanic, (**B**) chloroformic, and (**C**) methanolic extracts. We assessed viability using the MTT assay and expressed it as a percentage relative to untreated control cells. The data are shown as the mean ± SD from three independent experiments. We used one-way ANOVA for statistical analysis, followed by Tukey’s multiple comparison test. Asterisks denote significant differences when compared with both the untreated and vehicle control groups: *p* < 0.05 (*), *p* < 0.01 (**), *p* < 0.001 (***), and *p* < 0.0001 (****). Different letters indicate statistically significant differences (*p* < 0.05).

The effects of the hexanic, chloroformic, and methanolic extracts of *Tradescantia pendula* on U937 cell viability were evaluated after 24 h of exposure using the MTT assay (Figure 3).

The cells were exposed to increasing concentrations (6.25–200 μg/mL) of the hexanic, chloroformic, and methanolic extracts. Cell viability was assessed by the MTT assay and reported as a percentage relative to the untreated control cells. The data are shown as the mean ± SD from three separate experiments. Statistical analysis was carried out using one-way ANOVA followed by Tukey’s test for multiple comparisons. Asterisks denote significant differences compared with both the untreated control and the vehicle control (DMSO 0.1%): *p* < 0.05 (*), *p* < 0.01 (**), *p* < 0.001 (***), and *p* < 0.0001 (****). For the methanolic extract, concentrations of 50, 25, and 12.5 μg/mL (a) all exhibited significantly reduced viability compared with both the untreated control and the vehicle control (*p* < 0.05). Similarly, treatment with 6.25 μg/mL (b) was significantly different from both the untreated control and the vehicle control (*p* < 0.05).

Although the MTT assay is a widely used initial screening method for assessing cellular metabolic activity and viability, it does not distinguish among mechanisms of cell death. Therefore, future studies should incorporate complementary approaches to confirm the cytotoxic effects observed in this study, including the use of apoptosis and necrosis markers (such as Annexin V/propidium iodide staining, caspase activation assays, and lactate dehydrogenase release), as well as assessments of mitochondrial membrane potential, intracellular reactive oxygen species production, and alternative viability assays. These additional studies will provide a more comprehensive understanding of the mechanisms underlying the biological effects of *T. pendula* extracts.

The hexanic extract caused a noticeable, concentration-dependent change in cellular metabolic activity (see Figure 3A). Cell viability increased stepwise from 150 to 75 μg/mL and reached levels above 130% compared with the untreated controls. In contrast, viability dropped sharply at 50, 25, and 12.5 μg/mL, falling to about 20% of the control value. Statistical analysis showed significant differences between the untreated and vehicle controls, as well as among several treatment concentrations, especially at 100, 75, 50, 25, and 12.5 μg/mL (*p* < 0.01 to *p* < 0.0001).

The chloroformic extract also showed a biphasic response pattern (Figure 3B): cell viability rose from 150 to 75 μg/mL, attaining its maximum at 75 μg/mL. In contrast, exposure to 50 and 25 μg/mL substantially decreased metabolic activity, with viability dropping to near 20% of the control. Significant differences were found between the control groups and several treatment concentrations, with *p* values ranging from *p* < 0.05 to *p* < 0.0001.

The methanolic extract also showed concentration-dependent effects on U937 cells (Figure 3C): viability was nearly the same as the control at 200 and 150 μg/mL and increased at 100 and 75 μg/mL. In contrast, concentrations of 50, 25, and 12.5 μg/mL significantly reduced cell viability to about 20% of the control level (group a). Interestingly, treatment with 6.25 μg/mL partially recovered metabolic activity, with values only slightly above the 50% viability threshold (group b). Statistical analysis showed that both group a (50, 25, and 12.5 μg/mL) and group b (6.25 μg/mL) were significantly different from the untreated and vehicle controls (*p* < 0.05). In general, U937 cells were more sensitive to the extracts than HaCaT cells.

Each of the three extracts showed a non-monotonic concentration-response pattern, consisting of higher metabolic activity at intermediate concentrations and substantial decreases in viability at lower concentrations. This indicates that the response varies by cell type with respect to the bioactive metabolites in the *T. pendula* extracts.

### 2.7. Gas Chromatography (GC-MS)

GC-MS analysis of selected fractions from the chloroform extract was based on chromatographic resolution and the presence of well-defined peaks with high spectral similarity indices (≥90) and retention indices, allowing reliable compound identification. However, the identification is still considered tentative until the spectroscopic data have been confirmed. The compounds that have been tentatively identified are given in Table 6, Table 7 and Table 8.

The fractions 10, 12, and 27 were selected for GC-MS analysis based on the chromatographic profiles and biological activity observed during the initial screening.

The GC-MS analysis of fraction 10 (as shown in Table 6) showed a predominantly lipophilic character. This character is made up of linear and branched hydrocarbons, fatty and wax esters, long-chain ketones, fatty alcohols, and diols. This chemical composition is consistent with that normally found in epicuticular waxes and in the secondary storage lipids of vascular plants, organisms that have evolved to prevent desiccation and respond to environmental stressors [9,10].

**Table 6:** Fraction 10 of the chloroform extract.

Name	Rt [min]	RI	RI^Lit^	Area %	SI	Identification
Dodecane, 2,6,10-trimethyl-	7.4	1361	1365	0.12		MS+LRI
Pentadecane	8.0	1465	1500	0.17		MS+LRI
Heptadecanoic acid, ethyl ester	14.4	2074	2077	1.01	95	MS+LRI
Nonadecanoic acid, ethyl ester	16.2	2287	2277	0.39		MS+LRI
Hexacosane	18.0	2616	2600	0.66	93	MS+LRI
Phytyl octanoate	18.5	2710		0.49		MS+TLRI
Pentacosanal	18.8	2768	2738	0.16	92	MS+LRI
Octacosane, 2-methyl-	19.1	2831	2861	1.66	93	MS+LRI
2-Methyltriacontane	20.1	3041	3060	1.75		MS+LRI
Dotriacontane	21.1	3200	3200	4.2		MS+LRI
6-Methyl-dotriacontane	21.4	3248	3245	2.65		MS+LRI
Triacontane-1,30-diol	22.7	3408	3206 *	8.26		MS+LRI
16-Hentriacontanone	23.1	3452	3304	7.5		MS+LRI
4-Tritriacontanone	24.8	3614		17.53		MS+TLRI
Triacontyl butyrate	25.3	3655		1.61		MS+TLRI
Phytyl stearate	26.3	3722	3752	2.82	90	MS+LRI
Tetratriacontane-1,34-diol	27.8	3824		36.19		MS+TLRI

* From reference [11]; Rt refers to retention time; RI is the retention index calculated from a series of n-alkanes; RI^Lit^ is the retention index from the NIST 2023 database [12] or the NIST Chemistry WebBook 2026 [13]; SI stands for similarity index; where it is absent, means the compound is not in the equipment NIST database and the tentatively identification was made from its MS and LRI or TLRI; MS+LRI indicates that the identification was based on the mass spectrum and the linear retention index reported in the literature; MS+TLRI means the identification was based on the mass spectrum and the theoretical linear retention index, because no experimental LRI was found in the literature.

The linear and branched alkanes identified (namely, pentacosane, hexacosane, dotriacontane, and 6-methyl-dotriacontane), together with the very-long-chain alcohols and diols (specifically, triacontane-1,30-diol and tetratriacontane-1,34-diol), are characteristic constituents of the aliphatic cuticular wax fraction. These non-polar lipids are produced in epidermal cells through fatty acid elongation (FAE) complexes and deposited on the leaf surface, where they serve as an important hydrophobic barrier against desiccation and as the first physical defense against mechanical damage and penetration by fungal pathogens [14,15]. In *Tradescantia*, the waxes take the form of disordered microstructural platelets which, in addition to serving as a hydrophobic barrier against desiccation, also selectively reflect visible wavelengths and thus give rise to an optical sheen that offers protection against UV radiation [16,17].

On the other hand, the presence of 16-hentriacontanone (palmitone) confirms activation of the very-long-chain fatty acid decarboxylation biosynthetic pathway, a chemotaxonomic trait shared with other succulent monocots exposed to direct solar radiation [18].

Lipophilic esters derived from phytol (such as phytyl octanoate and phytyl stearate) have significant pharmacological value. Phytol is a diterpene alcohol released during senescence or as a result of chlorophyll turnover. To prevent potential cellular toxicity associated with lipids, plant cells esterify it with free fatty acids (stearate or octanoate) so it can be safely stored within the plastoglobules [19]. Free phytol and its esterified derivatives, obtained from the organic fractions of *T. pendula*, are responsible for the genus’s ethnomedicinal properties, as they have been shown to exert strong anti-inflammatory effects in vivo by reducing neutrophil responses and preventing the release of pro-inflammatory cytokines such as IL-1β and TNF-α [20]. They have also been widely reported to show antioxidant, antimicrobial, and cytotoxic activities via a number of biological pathways, including those involving the modulation of oxidative stress responses, and the identification of these metabolites supports the idea that oxygenated lipid derivatives may be more significant in producing the observed biological activities than the hydrocarbon fraction [21].

Finally, the same compounds were found in other plant species, like dotriacontane, hexacosane, and 16-hentriacontanone in *Tradescantia pallida* [17]; phytyl stearate, heptadecanoic acid ethyl ester, and pentadecane in *T. spathacea* [20]; and 16-hentriacontanone (palmitone), triacontane-1,30-diol, and octacosane in *Allium cepa* (Amaryllidaceae), a classic monocot model for the study of waxes [18].

**Table 7:** Fraction 12 of the chloroform extract.

Name	Rt [min]	RI	RI^Lit^	Area %	SI	Identification
3-Hexanone	3.4	823	795	0.25	95	MS+LRI
2-Hexanone	3.5	827	806	1.25	94	MS+LRI
Nonane	4.5	940	900	1.37	93	MS+LRI
Cyclohexane, (2-methylpropyl)-	4.8	979	988	2.24	91	MS+LRI
3,6-Dimethyldecane	5.9	1113	1129	0.38	90	MS+LRI
Undecane, 4-methyl	6.1	1152	1158	12.27		MS+LRI
Undecane, 3-methyl	6.2	1170	1169	1.08		MS+LRI
cis-2-Methyldecahydronaphthalene	6.4	1185	1184	6.07	94	MS+LRI
Dodecane, 5-methyl	6.8	1257	1255	14.73		MS+LRI
Tridecane, 4-methyl	6.9	1271	1268	5.22		MS+LRI
Tridecane, 3-methyl	7.4	1362	1372	12.86		MS+LRI
Tridecane, 2-methyl	7.5	1382	1366	1.83		MS+LRI
Methyl palmitate	12.8	1923	1921	2.57		MS+LRI

Rt: retention time; retention index (RI) is calculated using a homologous series of n-alkanes; RI^Lit^ is the retention index from the NIST 2023 database [12] or the NIST Chemistry WebBook 2026 [13]; SI stands for similarity index; where it is absent, means the compound is not in the equipment NIST database and the tentatively identification was made from its MS and LRI or TLRI; MS+LRI indicates that the identification was based on the mass spectrum and the linear retention index reported in the literature.

What stands out in the GC-MS analysis of fraction 12 is the high abundance of medium-chain volatile hydrocarbons, short-chain aliphatic ketones, and low-molecular-weight lipid esters. The compounds known as 3-hexanone and 2-hexanone are formed as a result of the oxidative catabolism of unsaturated lipids. Although they occur alongside green-leaf compounds from the lipoxygenase pathway, these ketones are generally produced by alternative enzymatic decarboxylation pathways during wilting or cellular stress [22].

In contrast, compounds such as methyl palmitate, nonane, and branched alkanes represent a significant finding that supplements the metabolic map of *T. pendula*. Although the classical literature on this species has mainly focused on polar compounds, these volatile and semi-volatile analytes characterize the plant’s “volatilome”. The homologous series of intermediate-chain alkanes (from C9 to C13)—including both straight-chain forms and their branched isomers—plays an important biophysical role in plants adapted to leaf succulence. These hydrocarbons function as solvents and help maintain the fluidity of internal lipid membranes, thus preventing the crystallization of structural fatty acids when exposed to extreme temperature changes in tropical climates [23]. Moreover, the presence of methyl palmitate opens a new line of methodological and pharmacological inquiry. If its origin can be excluded as a transesterification artifact—that is, one caused by the use of alcoholic solvents during processing—then this methyl ester must be regarded as an endogenous metabolite derived from the saturated fatty acid synthesis pathway. The buildup of this compound in cells is of biomedical importance, as it is consistent with the plant’s anti-inflammatory activity observed in in vivo studies; this effect is due to the known ability of fatty esters to reduce pro-inflammatory signaling cascades and inhibit nitric oxide production in macrophages [20].

**Table 8:** Fraction 27 of the chloroform extract.

Name	Rt [min]	RI	RI^Lit^	Area %	SI	Identification
Succinimide	6.3	1171	1187	1.50	96	MS+LRI
5-Methoxypyrrolidin-2-one	6.6	1234		1.38	92	MS+TLRI
Benzothiazole	7.1	1312	1271	1.01	92	MS+LRI
2-Cyclohexen-1-one, 4-hydroxy-3,5,5-trimethyl-4-(3-oxo-1-butenyl)-	12.3	1884	1802 *	1.73	92	MS+LRI
Isophytol	12.7	1914	1922	54.37		MS+LRI
Palmitic acid	14.0	2038	2003	25.39		MS+LRI

* From reference [24]; Rt: retention time; RI: retention index calculated from a homologous series of n-alkanes; RI^Lit^: retention index from the NIST 2023 database [12] or the NIST Chemistry WebBook 2026 [13]; SI: similarity index; where it is absent, means the compound is not in the equipment NIST database and the tentatively identification was made from its MS and LRI or TLRI; MS+LRI indicates that the identification was based on the mass spectrum and the linear retention index reported in the literature; MS+TLRI indicates that the identification was based on the mass spectrum and the theoretical linear retention index, because no experimental LRI was found in the literature.

The GC-MS analysis of fraction 27 from *Tradescantia pendula* revealed a highly specialized molecular composition comprising nitrogen- and sulfur-containing heterocyclic derivatives, an isoprenol alcohol, and free fatty acids. The co-elution of these compounds is consistent with the use of a mobile phase of intermediate polarity, which promotes the elution of amphipathic substances and intracellular lipid components originating from the cytoplasmic and chloroplastic compartments. Because no existing publications specifically classify these molecules in *T. pendula*, the spectrum is presented here as a new account of the plant’s internal metabolic matrix. This level of complexity reflects inputs from both primary and secondary plant metabolism, as well as possible biotransformations caused by the endophytic microflora or by thermal degradation during chromatographic injection [25].

A key feature defining the chemical character of this fraction is its clear quantitative homogeneity. Across the entire elution profile of the fraction, isophytol, a diterpene alcohol, is the most abundant compound at 54.37%, followed by the saturated fatty acid palmitic acid at 25.39%. Together, these two components account for 79.76% of the chromatographic area, indicating that the silica gel fractionation has successfully and highly selectively isolated the plant’s cellular lipid core. The high level of isophytol—a structural isomer of phytol—can be explained biochemically by metabolic turnover processes in the chloroplast, where it functions as a stable hydrophobic intermediate derived from the cleavage and regulated degradation of the phytyl chains of leaf chlorophylls. By contrast, the 25.39% content of palmitic acid (C16:0) serves as the main lipid substrate for cell membranes. The extensive co-elution of this free fatty acid with the structural diterpenoid gives the fraction its hydrophobic properties, a feature consistent with the antioxidant and cytoprotective activities generally observed in non-polar fractions from this genus [20].

Several heterocyclic compounds were identified, including benzothiazole (0.47%), succinimide (0.5%), and 5-methoxypyrrolidin-2-one (0.58%). Because benzothiazole and other heterocycles are part of bioactive scaffolds with antimicrobial, anticancer, and pharmacological activities, especially in synthetic and semi-synthetic drugs, their direct role in the extract’s biological activity remains uncertain [26,27].

## 3. Discussion

This study provides evidence of the biological activity and phytochemical composition of the hexane, chloroform, and methanol extracts of *Tradescantia pendula*. Phytochemical screening detected flavonoids, tannins, triterpenes, and glycosides, metabolites typically associated with antioxidant and antimicrobial properties in medicinal plants [28,29,30]. In the Commelinaceae family, which includes *Tradescantia zebrina* (*T. pendula*) and *Commelina benghalensis*, similar phytochemical profiles have already been reported, indicating the presence of bioactive secondary metabolites in this botanical group [31,32].

GC-MS analysis of *Tradescantia pendula* reveals a coordinated mechanism for multi-target bioactivity that underlies its ethnomedicinal value. The fraction known as 10, with a high content of tetratriacontane-1,34-diol (36.19%) and 4-tritriacontanone (17.53%), forms a lipophilic layer that serves as the first line of biological defense; these long-chain waxes not only reduce desiccation but also prevent the mechanical adhesion of fungal spores, thus limiting early pathogen colonization. At an intermediate level, the first fraction exhibits a defensive volatilome consisting mainly of branched alkanes—for example, 5-methyldodecane (14.73%), 3-methyltridecane (12.86%), and 4-methylundecane (12.27%)—compounds that function as chemical signals to alert cells and trigger immunity. Ultimately, fraction 27 contains the pharmacological essence of the extract because isophytol (54.37%) and palmitic acid (25.39%) co-elute extensively; this main lipid matrix shows anti-inflammatory and systemic cytoprotective properties by inhibiting the biosynthesis of pro-inflammatory cytokines and reducing tissue oxidative stress. Moreover, this hydrophobic substrate acts as a cellular carrier for several highly specialized heterocyclic trace compounds, such as benzothiazole and pterolactam; the fused rings in these molecules contribute to the extract’s overall bioactivity by directing effects toward apoptosis in biotic vectors and phytopathogenic fungi [33,34].

The biological activities observed in similar plant extracts are more likely attributable to oxygenated metabolites, such as phytol-related compounds and fatty acid derivatives, rather than to simple hydrocarbons or trace volatile aromatic compounds; the presence of multiple antioxidant and bioactive lipid compounds suggests that synergistic effects may account for the antimicrobial activity [35].

Because the tentatively identified compounds show high spectral similarity (≥90) and similar linear retention indices, and because similar compounds have been reported to have biological activity, the extract may be a promising source of bioactive molecules with anti-inflammatory properties. This is especially important for potential uses, such as dermal formulations, where both antimicrobial and anti-inflammatory compounds could improve skin protection. However, further studies using isolated fractions and specific bioassays are needed to confirm each compound’s individual contribution [36].

The antimicrobial tests revealed inhibitory effects primarily against Gram-negative bacteria, specifically *Escherichia coli* and *Pseudomonas aeruginosa*; however, no significant activity was observed against Gram-positive strains under the experimental conditions. The differing susceptibility of Gram-positive and Gram-negative bacteria may be due to differences in their cell envelope structure, membrane permeability, and intrinsic resistance mechanisms [37,38]. Although the minimum inhibitory concentrations were relatively high compared with those of conventional antibiotics, the extract still caused considerable decreases in bacterial viability at sublethal concentrations, indicating compounds that interfere with bacterial growth or membrane stability [39,40,41].

A reference antibiotic was not included as a positive control because the primary objective of this study was to screen the antimicrobial potential of *T. pendula* preliminary extracts rather than compare their efficacy with clinically used antimicrobial agents. Such comparisons are methodologically limited because crude plant extracts are complex mixtures of secondary metabolites with distinct physicochemical properties, diffusion properties, and mechanisms of action, whereas antibiotics are highly purified compounds with well-defined biological targets. Consequently, comparisons based solely on inhibition zone diameters or MIC values may lead to misleading interpretations. Instead, the extracts were evaluated under identical experimental conditions to reliably assess their relative antimicrobial activity and identify promising candidates for future phytochemical characterization and validation studies.

The antibacterial activity observed in the present study varied markedly among bacterial isolates, as reflected by the different MIC values obtained. Such differences are expected in crude plant extracts and have been widely reported in the genus *Tradescantia*, where factors including plant species, extraction solvent, phytochemical composition, and bacterial susceptibility influence antimicrobial activity. A comprehensive review by [42] summarized MIC values that generally ranged from 2.5 to 10 mg/mL for *Tradescantia* extracts, highlighting considerable variability among studies. In this context, the MIC of 0.404 mg/mL obtained against some isolates in the present study compares favorably with previously reported values, whereas the higher MIC observed for other isolates suggests strain-dependent susceptibility.

These findings are consistent with previous reports on *Tradescantia* species [30]. Reported MIC values between 5 and 10 mg/mL for extracts from several Commelinaceae species, including *T. zebrina* (*T. pendula*), and concluded that antibacterial activity varied according to plant species and bacterial strain. Similarly, [4] confirmed the antibacterial potential of *T. zebrina* (*T. pendula*), attributing its activity to phenolic compounds, flavonoids, and tannins. Thus, these findings support the hypothesis that the antibacterial activity of *Tradescantia* extracts results from the combined or synergistic action of multiple phytochemicals rather than a single bioactive constituent. Therefore, although crude plant extracts generally exhibit higher MIC values than purified antimicrobial agents, their biological relevance should be interpreted in the context of complex phytochemical mixtures, highlighting *Tradescantia*’s potential as a source of antimicrobial compounds.

Comparing the toxicity of the chloroform extract of *Tradescantia pendula* in the *Artemia* model with the literature on species in the same genus and family reveals notable differences that highlight this species’ unique bioactivity. Our findings show exceptionally high acute toxicity for the chloroform extract, with an LC_50_ of 23.12 μg/mL. This value is significantly lower than that reported for related species. For example, studies on *Tradescantia spathacea* have reported LC_50_ values for the chloroformic and hydrophilic fractions ranging from 200 to 350 μg/mL. Meanwhile, *T. pallida* extracts typically exceed 167 μg/mL in the same model [43]. Compared with Meyer et al. [44], the chloroformic and methanolic extracts showed toxicity similar to berberine chloride, while the hexanic extract was comparable to strychnine sulfate, indicating strong toxicity for all three extracts. This disparity suggests that *T. pendula* possesses a unique phytochemical profile, possibly enriched in lipophilic secondary metabolites (terpenoids, alkaloids, or steroids), and exhibits a much more pronounced systemic lethal activity than its congeners [45].

The cytotoxicity assays demonstrated distinct responses across the cellular models evaluated. Notably, the three extracts exhibited cell type-dependent effects, with U937 monocytic cells showing greater sensitivity than HaCaT keratinocytes. While the chloroformic and methanolic extracts produced limited reductions in HaCaT viability, the hexanic extract induced a marked decrease in metabolic activity at higher concentrations. In contrast, all three extracts significantly affected U937 cells, suggesting that immune-related cells may be more susceptible to the bioactive constituents of *T. pendula*. An interesting observation was the non-monotonic concentration-response pattern detected in both cell lines. In HaCaT cells, the hexanic extract produced the most pronounced effect, reducing viability at higher concentrations while increasing MTT reduction at intermediate concentrations. Likewise, in U937 cells, all three extracts promoted enhanced metabolic activity at intermediate concentrations (75–150 μg/mL), followed by marked reductions in viability at lower concentrations (12.5–50 μg/mL). Such biphasic responses have been described for numerous phytochemicals that modulate mitochondrial metabolism, redox homeostasis, and cellular stress signaling pathways [4,30,40,41,42].

This behavior may be compatible with a hormetic-like response, a biological phenomenon characterized by adaptive cellular activation at specific exposure levels and inhibitory or cytotoxic effects at others [40,41,42]. Similar responses have been reported for plant-derived terpenoids and lipophilic secondary metabolites, which can stimulate mitochondrial activity and intracellular reducing capacity without necessarily increasing cell proliferation [4,30].

However, interpreting MTT-based responses requires caution. Tetrazolium reduction assays primarily reflect cellular metabolic activity and intracellular reducing capacity rather than direct measurements of cell number or proliferation. Consequently, increased absorbance values may indicate transient metabolic stimulation or redox adaptation rather than true proliferative effects. Therefore, the increase in MTT signal observed in this study should be interpreted as enhanced metabolic activity rather than definitive evidence of cell proliferation. This interpretation is consistent with previous methodological studies indicating that MTT assays can be influenced by mitochondrial activity, intracellular NAD(P)H production, and redox-active phytochemicals.

The greater sensitivity observed in U937 cells may reflect intrinsic differences in cellular metabolism and susceptibility to oxidative and membrane-active compounds. Monocytic cells possess distinct bioenergetic requirements and inflammatory signaling pathways compared with epithelial-derived cells, which may render them more responsive to lipophilic metabolites such as terpenoids, phytol derivatives, and fatty acid esters. Previous studies have shown that these compound classes can alter mitochondrial function, membrane permeability, redox balance, and signaling pathways involved in cell survival in immune-related cellular models [24,30,32].

Although GC tentatively identified several compounds by MS, the contribution of individual metabolites to the observed biological activities remains unclear due to the extract’s chemical complexity. Additionally, the antioxidant and cytotoxic effects observed may result from synergistic or antagonistic interactions among multiple constituents [46,47,48]. Therefore, further studies involving bioactivity-guided fractionation, structural confirmation of metabolites, quantification of ROS, assessment of apoptosis markers, analysis of mitochondrial membrane potential, and complementary viability assays are needed to better characterize the biological properties and mechanisms of action of *T. pendula* extracts.

Overall, the results suggest that *Tradescantia pendula* extracts contain bioactive metabolites capable of exerting antioxidant, antimicrobial, and cell type-dependent cytotoxic effects. The differential responses observed between HaCaT and U937 cells, together with the marked toxicity detected in the *Artemia salina* model and the identification of lipophilic metabolites by GC–MS could support the biological activity of this species and highlight its potential use as a source of natural products beneficial to human health. However, further scientific studies are needed to reach a more conclusive answer.

## 4. Materials and Methods

### 4.1. Plant Material and Extraction

Samples of *Tradescantia pendula* were collected in September 2023 from the Francisco Peláez Roldán Ethnobotanical Garden located in the city of San Andrés Cholula, Puebla, Mexico. The National Laboratory for Plant Identification and Characterization at the University Center for Biological and Agricultural Sciences (CUCBA) of the University of Guadalajara, Mexico identified the species; the samples were deposited in the herbarium under accession number IBUG 214426.

We obtained fresh plants weighing 6.664 kg. We manually defoliated the plants and cut the stems to approximately 2 cm. We carried out this procedure with gloves to prevent potential reactions that could affect the research results and, as a health precaution, to reduce the risk of allergies. Subsequently, the leaves and stems were dried in a well-ventilated, shaded area, following the protocol described by Juárez et al. [49].

The dried plant material was placed in an Erlenmeyer flask and macerated sequentially with hexane, chloroform, and methanol. It was shaken occasionally for 72 h at room temperature. The macerated plant material was filtered through a long-stemmed funnel with a cotton plug. The solvents were then evaporated using a rotary evaporator (R-100, BÜCHI Labortechnik AG, Flawil, Switzerland) at 35 °C. The extracts were then transferred to amber glass vials, dried in a desiccator until completely dry, covered, and stored in the refrigerator at 4 °C [50].

### 4.2. Phytochemical Screening

Aliquots of approximately 20 mg of each extract, dissolved in 1 mL of hydroethanolic solution (70% *v*/*v*), J.T. Baker, were used to test the chemical groups listed below. All tests were conducted using Domínguez’s method [51].

### 4.3. Alkaloids

An ethanol extract suspension in 1% HCl J.T. Baker was prepared. After filtration, aliquots were used to detect alkaloids using the Mayer, Wagner, and Dragendorff-Meyer reagents from J.T. Baker (Phillipsburg, NJ, USA). If a precipitate was observed, the test was considered positive for alkaloids.

### 4.4. Test for Saponins

The ethanol extract (20 mg) was mixed with hot distilled water and stirred vigorously for 3–5 min. The test was considered positive when stable foam formed and persisted for at least 30 min.

### 4.5. Triterpene Assay

1 mL of acetic anhydride was mixed with 20 mg of ethanol extract from each species. After 5 min, 1–2 drops of concentrated H_2_SO_4_ (Meyer, Mexico City, Mexico) were added, and the mixture was allowed to stand for 1 min. The test was considered positive if a reddish, pink, purple, green, or blue ring was observed at the interface.

### 4.6. Tannin Tests

The ethanol extracts were filtered and tested with two or three drops of 10% FeCl_3_ (J.T. Baker) and two or three drops of gelatin reagent. If a blackish-blue precipitate was observed, the test was considered positive for hydrolyzable or gallic tannins; if the precipitate was brown or green, it indicated condensed tannin or catechol derivatives.

### 4.7. Assays for Flavonoids

The Shinoda test was performed by adding a small amount of amalgamated Mg (Meyer) and 2 drops of HCl concentrate (J.T. Baker) to the extract aliquot. The development of a red or magenta color indicated the presence of dihydroflavonols, flavanols, or flavanones, whereas the appearance of an orange color indicated the presence of flavanones.

Another test used was the Pew test. The extracts were mixed with 0.1 g of metallic Zn (Sigma-Aldrich, Toluca, Mexico) and two drops of 5N HCl. If the solution turned reddish, purple, or cherry red, the test was considered positive for dihydroflavonoid. In contrast, the presence of dihydrochalcones, flavanones, and other flavonoids was indicated by the appearance of a pink or brown color. The NaOH (J.T. Baker) test was also performed to detect flavonoids; a yellowish or orange color indicates a positive result when 2 drops of 5% sodium hydroxide solution are added.

### 4.8. Cyanogenic Glycoside Assay

A filter paper impregnated with the Guignard reagent was placed in a water bath for 30 min, then dried before exposure to the extracts. The test was considered positive when the paper turned red or pink.

### 4.9. Cardiotonic Glycosides and Sesquiterpene Lactones Tests

To an aliquot of each ethanol extract, two or three drops of the Baljet reagent were added, and the appearance of orange or dark red colorations was considered indicative of a positive test.

### 4.10. Evaluation of the Antimicrobial Activity of Extracts

#### Activity Screening

The antimicrobial activity of *Tradescantia pendula* extracts was evaluated using fractions obtained with solvents of different polarity (hexane and chloroform). The dried extracts were resuspended in dimethyl sulfoxide (DMSO) and subsequently diluted in a series to obtain final concentrations of 103,400; 51,700; 25,800; 12,900; 6400; 3200; 1615; 808; 404; and 202 μg/mL. Antimicrobial activity was determined using the spot (drop) method against pathogenic bacterial strains including *Escherichia coli* (2537 and 3002), *Pseudomonas aeruginosa* (3134), *Staphylococcus aureus* (QO1), and *Staphylococcus epidermidis* (13538). These microorganisms were isolated from clinical samples with consent at the “Hospital del Niño Jesús” in the city of San Miguel de Tucumán, Province of Tucumán, Argentina. The bacterial strains were inoculated at 10^6^ CFU/mL in Müller-Hinton Agar medium. After applying the extracts at different concentrations, the plates were incubated for 24 h at 37 °C. The appearance of inhibition halos was interpreted as evidence of antimicrobial activity.

### 4.11. Effect on Bacterial Viability at Inhibitory Concentrations

To further evaluate the antimicrobial activity and analyze the effects of the detected sub-inhibitory concentrations (25,800 μg/mL, 404 μg/mL), a dilution immediately below the minimum inhibitory concentration (MIC) determined in the screening test was used. This stage aimed to determine the cell viability of the indicator microorganisms that showed sensitivity to the extracts.

To this end, Müller-Hinton liquid media were prepared and inoculated with bacterial suspensions adjusted to an initial concentration of 10^6^ CFU/mL, to which the corresponding extract was added at a concentration immediately below the MIC. The systems were incubated for 24 h at 37 °C, after which they were reseeded onto Müller-Hinton agar plates. After a further 24 h of incubation at 37 °C, bacterial colony formation was evaluated, and the growth obtained was compared with a negative control prepared identically to the sample but without the extract to determine the reduction in the viable population under sublethal conditions.

### 4.12. Brine Shrimp Assay

Brine shrimp cysts from *Artemia* spp. (Cystemia^®^ from Artemias S de RL de CV, harvested from Great Salt Lake, UT, USA) were hatched in artificial seawater prepared with 30 g/L of commercial sea salt dissolved in distilled water. The cysts were incubated at 25 °C under a 24 h photoperiod. We transferred 24 h-old nauplii to test tubes using a pipette. The nauplii were exposed to a *T. pendula* chloroformic extract, dissolved in DMSO, at concentrations of 50, 75, 100, 150, 250, 500, 1000, and 2000 μg/mL for 24 h. Artificial seawater with DMSO was used as the blank control. After 24 h, dead nauplii were counted. The larvae were considered dead when no movement was observed after 10 s. All experiments were conducted in triplicate. Toxicity was estimated by calculating the mean lethal concentration (LC_50_) after a 24 h incubation period. The LC_50_ value was determined by plotting extract concentration against the percentage of dead nauplii and fitting the data to the Probit model. The values obtained were compared with the standard classification [49].

### 4.13. Cytotoxicity Test

The in vitro cytotoxic activity of the hexane, chloroform, and methanol extracts of *Tradescantia pendula* was evaluated using the human epidermal keratinocyte cell line HaCaT and the human monocytic cell line U937. HaCaT cells were cultured in Dulbecco’s Modified Eagle Medium (DMEM), whereas U937 cells were maintained in RPMI-1640 medium. Both culture media were supplemented with 20% fetal bovine serum (Biowest, Nuaillé, France), 1% L-glutamine, and 1% antibiotic-antimycotic solution (Gibco, CTR Scientific, Monterrey, Nuevo León, Mexico). Cells were incubated at 37 °C in a humidified atmosphere with 5% CO_2_ until they reached approximately 60–70% confluence. For cytotoxicity assays, cells were seeded into 96-well plates and incubated overnight to allow stabilization. Subsequently, the hexane, chloroform, and methanol extracts were added at final concentrations of 3.12, 6.25, 12.5, 25, 50, 75, 100, 150, and 200 μg/mL, and the mixture was incubated for 24 h. Dimethyl sulfoxide (DMSO, 0.1%) was used as the vehicle control.

Cell viability was determined using the MTT colorimetric assay based on the reduction of 3-(4,5-dimethylthiazol-2-yl)-2,5-diphenyltetrazolium bromide to insoluble formazan crystals by metabolically active cells. Briefly, 50 μL of MTT solution (5 mg/mL) was added to each well 2.5 h before the end of incubation. Following incubation, absorbance was measured at 570 nm using an Epoch microplate reader (BioTek, Winooski, VT, USA). Cell viability was expressed as a percentage relative to untreated control cells. All experiments were performed in triplicate, and results are presented as mean ± standard deviation (SD). For the chloroformic extract, half-maximal inhibitory concentration (IC_50_) values were estimated by non-linear regression analysis of the dose–response curves using GraphPad Prism version 10 (GraphPad Software, San Diego, CA, USA).

### 4.14. Column Chromatography

A column chromatography procedure was carried out using a silica column with a diameter-to-height ratio of 1:20. The required amount of silica was calculated using 22.72 g of chloroformic extract, and the column was packed with 568.15 g of silica gel (60–230 mesh). The previously silica-adsorbed extract was loaded onto the column and eluted with hexane-chloroform (80:20) at a flow rate of 1 drop per second, with the mobile-phase polarity gradually increased to 100% methanol.

The collected fractions were analyzed by thin-layer chromatography to determine which fractions could be pooled. Subsequently, the solvents were evaporated to obtain the corresponding fractions.

### 4.15. Gas Chromatography

Gas chromatography-mass spectrometry analysis of the chloroform extracts was performed using a SHIMADZU Nexis GC-2030 gas chromatograph, coupled to a QP2020NX mass spectrometer as a detector (Shimadzu Corp., Kyoto, Japan).

Separation was carried out on a SH-I-5MS capillary column, with a 0.25 μm thickness and a 30 m length. The injector temperature was set to 250 °C, and samples were injected in split mode with a 1 μL injection volume.

The oven temperature program started at 60 °C and was held for 2 min, then increased at 30 °C/min to 200 °C and was held for 5 min, followed by a final increase at 20 °C/min to 325 °C and a 10 min hold. Helium was used as the carrier gas at a column flow rate of 1.0 mL/min.

Linear retention indices (LRI) were calculated from the n-alkane homolog series (C8-C40). Tentative identification of the detected compounds was performed by comparing their mass spectra with those in the NIST Mass Spectral Library and the Wiley Registry of Mass Spectral Data. Tentative identification was based on a similarity index (SI) ≥ 90 and an LRI match within LRI ± 40, using literature (LRI) or theoretical (TLRI) values if not found in the literature.

## 5. Conclusions

The hexane, chloroform, and methanol extracts of *Tradescantia pendula* exhibited antimicrobial, toxic, and cell-type-dependent cytotoxic activities, which may be associated with lipophilic metabolites identified by GC–MS analysis, including phytol-related compounds and fatty acid esters. Phytochemical screening confirmed the presence of flavonoids, tannins, triterpenes, and glycosides, supporting this species’ biological potential.

The antimicrobial activity observed against Gram-negative bacteria, together with the toxicity detected in the *Artemia salina* model and the differential responses observed in HaCaT and U937 cells, indicates that *T. pendula* contains bioactive constituents capable of modulating cellular viability and metabolic activity. Notably, several plant-derived extracts have been associated with antioxidant, anti-inflammatory, antimicrobial, cytotoxic, and redox-modulating properties. The differential sensitivity observed between epithelial (HaCaT) and monocytic (U937) cells, as well as the non-monotonic concentration-response patterns detected in vitro, suggest that the biological effects of *T. pendula* extracts may involve complex interactions with cellular metabolic and redox pathways.

Therefore, the correlation between the phytochemical composition tentatively identified in the present study and the biological activities observed could support the ethnopharmacological relevance of *T. pendula*. Nevertheless, further investigations involving bioactivity-guided fractionation, compound isolation, structural confirmation, mechanistic studies, and in vivo evaluations are required to validate its biological properties and to identify the metabolites responsible for the observed activities.

## Figures and Tables

**Table 1:** Performance of plant extracts for *Tradescantia pendula*.

Extract	Total Mass (g)	Yield (%)
Hexane	3.06	0.54
Chloroform	22.76	4.04
Methanol	10.27	1.82

**Table 2:** Results of preliminary phytochemical analyses of *Tradescantia pendula*.

Metabolites	Chloroform Extract
Alkaloids	−
Saponins	−
Triterpenes	+
Tannins	+
Flavonoids	+
Glycosides	+

(−) Absence of metabolites, (+) presence of metabolite.

**Table 3:** Screening of antimicrobial effects of extracts from *Tradescantia pendula*.

Microorganism	MIC (μg/mL)	Extract
*Escherichia coli* 2537	25,800	Chloroform
*Escherichia coli* 2537	>103,400	Hexane
*Escherichia coli* 3002	404	Chloroform
*Escherichia coli* 3002	>103,400	Hexane
*Pseudomonas aeruginosa* 3134	404	Chloroform
*Pseudomonas aeruginosa* 3134	51,700	Hexane

**Table 4:** Effect on viability of *Tradescantia pendula*.

Strain	Treatment (Type and Concentration)	Final Viability (CFU/mL)	Reduction in Viability Compared to the Control (%)
*Escherichia coli* 2537	Chloroform 25,800 μg/mL	1.20 × 10^6^	95.2
*Escherichia coli* 3002	Chloroform 404 μg/mL	5.30 × 10^6^	82.9
*Pseudomonas aeruginosa* 3134	Chloroform 404 μg/mL	3.40 × 10^6^	64.2
*Pseudomonas aeruginosa* 3134	Hexane 51,700 μg/mL	4.50 × 10^6^	52.6

## Data Availability

The data presented in this study are available on request from the corresponding authors, in accordance with university policies.

## Abbreviations

The following abbreviations are used in this manuscript:

ANOVA	Analysis of Variance
CFU	Colony-Forming Units
CUCBA	University Center for Biological and Agricultural Sciences
DMSO	Dimethyl Sulfoxide
GC-MS	Gas Chromatography-Mass Spectrometry
HaCaT	Human Adult High Calcium Low Temperature cells
IC_50_	Half Maximal Inhibitory Concentration
LC_50_	Half Lethal Concentration
MIC	Minimum Inhibitory Concentration
MTT	3-(4,5-dimethylthiazol-2-yl)-2, 5-diphenyltetrazolium bromide
NAD(P)H	Reduced Nicotinamide Adenine Dinucleotide Phosphate
ROS	Reactive Oxygen Species
Rt	Retention time
SI	Similarity index
WHO	World Health Organization
